# Corticosterone enhances CSD susceptibility via glucocorticoid receptor activation in familial hemiplegic migraine 1 Cacna1a knock-in mice

**DOI:** 10.1186/1129-2377-14-S1-P85

**Published:** 2013-02-21

**Authors:** R Shyti, K Eikermann-Haerter, OC Meijer, SH van Heiningen, L De Groote, MD Ferrari, C Ayata, AMJ van den Maagdenberg, EA Tolner

**Affiliations:** 1 Dept of Human Genetics, Leiden Univ. Medical Center, Leiden, Netherlands; 2 Neurovascular Research Laboratory, Dept of Neurology, Massachusetts General Hospital, Harvard Medical School, Boston, USA; 3 Dept of Endocrinology, Leiden Univ. Medical Center, Leiden, Netherlands; 4 Dept of Neurology, Leiden Univ. Medical Center, Leiden, Netherlands; 5 Dept of Neurology,, Netherlands; 6 Dept of Human Genetics, Leiden Univ. Medical Center, Leiden, Netherlands; 7 Dept Neurology, Leiden Univ. Medical Center, Leiden, Netherlands

## Introduction

FHM1 mutant mice carrying the R192Q gain-of-function mutation in CaV2.1 (P/Q-type) calcium channels display enhanced glutamatergic transmission and increased propensity for cortical spreading depression (CSD;1,2). Corticosteroids released after stress also enhance glutamatergic transmission but the relationship between stress and migraine is not well understood.

## Objectives

We aimed to investigate the acute effects of corticosterone and the role of GR activation on CSD susceptibility in FHM1 R192Q knock-in mice.

## Methods

Corticosterone (20 mg/kg) or vehicle was injected subcutaneously 4 hours before CSD frequency recordings were carried out in FHM1 R192Q mice. A subgroup of mice was injected with the glucocorticoid receptor antagonist mifepristone 50 minutes before corticosterone/vehicle injection.

## Results

Corticosterone injection increased CSD frequency in FHM1 mice compared to vehicle-injected controls but not in wild-types. Pretreatment with mifepristone reduced CSD frequency to the level of vehicle-injected controls. Baseline corticosterone plasma levels were similar in WT and FHM1 mice, while 3 hours after corticosterone administration corticosterone plasma levels were strongly elevated to comparable levels in both WT and FHM1 mice.

## Conclusion

These data suggest that combined effects of glucocorticoid receptor activation and the FHM1 R192Q CaV2.1 gain-of-function mutation on excitatory neurotransmission may play a role in proposed effects of stress on migraine attacks.
